# Therapeutic interfering particles against HIV: molecular parasites reducing viremia

**DOI:** 10.1038/s41392-024-02001-0

**Published:** 2024-10-14

**Authors:** Henning Gruell, Stanley Odidika, Philipp Schommers

**Affiliations:** 1 Institute of Virology, Faculty of Medicine and University Hospital Cologne, University of Cologne, Cologne, Germany; 2 Department I of Internal Medicine, Faculty of Medicine and University Hospital Cologne, University of Cologne, Cologne, Germany; 3 Center for Molecular Medicine Cologne (CMMC), University of Cologne, Cologne, Germany; 4 German Center for Infection Research (DZIF), Partner Site Bonn-Cologne, Cologne, Germany

**Keywords:** Gene delivery, Infectious diseases, Infectious diseases

In a recent article in *Science*, Leon Weinberger and colleagues report on successful proof-of-concept studies of a novel strategy to control HIV-1 infection by using interfering viral particles.^1^ This approach radically differs from small-molecule antiretroviral drugs that can prevent disease progression and transmission by inhibiting viral replication but require life-long use.

Conceptually, the work derives from decades-old observations of defective viral particles impeding replication of influenza viruses and other viral pathogens.^2^ Defective interfering particles (DIPs) can develop as a consequence of erroneous viral replication. While DIPs resemble their ancestors, they differ from intact viruses through genome alterations that prevent autonomous replication. However, in cells co-infected with a DIP and intact virus, DIPs can essentially become a molecular parasite that hijacks the viral replication machinery and promotes the production of defective over intact virions (e.g., due to preferential replication of the shorter DIP genome). Indeed, defective viral genomes can be used to exert antiviral activity in vivo and DIPs with a basic reproductive ratio (R_0_) > 1 have been proposed as long-acting treatment options (termed “therapeutic interfering particles” or TIPs).^1,3^ Although HIV-1 replication is notoriously error-prone, however, naturally occurring HIV-derived DIPs that could serve as blueprints for HIV-TIP development have not previously been described.

Based on in silico predictions of DIP growth characteristics, Pitchai and Tanner et al. ^1^ performed nondilutive long-term HIV-1/CD4 T cell co-cultures. After > 70 days of culture, they isolated proviral HIV-1 DNA with a ~ 2.5 kb deletion ( ~ 25% of the HIV-1 genome) in the *pol-vpr* region. Indicating bona fide DIP characteristics, the defective genome required HIV-1 protein expression in trans for replication (conditional replication) and interfered with HIV-1 production. Further genetic modifications that included the reinsertion of a deleted segment and ablated expression of additional viral proteins resulted in different variants with genomes as short as ~ 5.5 kb (55% of HIV-1; Fig. 1a). In fulfillment of the proposed criteria for HIV-TIPs, mutations in these variants were associated with an in vitro R_0_ > 1 in addition to conditional replication and HIV-1 replication interference (Fig. 1b). Finally, HIV-TIPs were demonstrated to establish latency in transduced cells in vitro that could be reversed by cessation of antiretroviral therapy and HIV rebound.

**Fig. 1:**
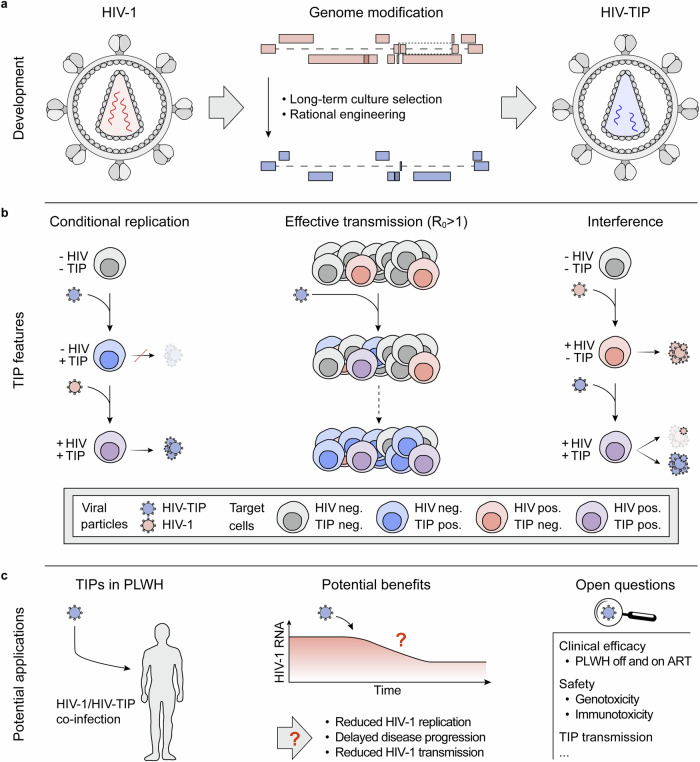
Therapeutic interfering particles (TIPs) for HIV-1 infection. **a** Development of HIV-1-based TIPs by genome editing. HIV-1 RNA deletions, mutations, and modifications included changes selected for in long-term culture and introduced through rational engineering. **b** Key characteristics of HIV-TIPs. (i), Dependence on HIV-1 co-infection for replication (conditional replication; left). (ii), Effective production and cellular transmission enabling sustained effects (R_0_ > 1; center). (iii), Interference with HIV-1 replication reducing production of HIV-1 particles (right). **c** Potential use of HIV-TIPs in people living with HIV (PLWH). Effective reduction of viremia through HIV-TIP interference may delay disease progression and reduce HIV-1 transmission. Open questions requiring further research include clinical efficacy, safety, and the potential for HIV-TIP transmission

To determine antiviral TIP efficacy in vivo, an optimized HIV-TIP and a simian immunodeficiency virus-adapted variant (SIV-TIP) were evaluated in murine and non-human primate (NHP) models of infection, respectively. In humanized mice, HIV-TIP administration five days after HIV-1 challenge resulted in reduced levels of HIV-1 viremia. These findings were extended to an infant rhesus macaque model in which infection with a chimeric simian/human immunodeficiency virus (SHIV) typically results in rapid disease progression and an AIDS-like condition within a few months. While peak viremia after SHIV challenge was overall similar in NHPs pre-treated with SIV-TIPs and controls, TIP-treated animals subsequently exhibited a reduced viral set point (3 to 5 log_10_ reduction in SHIV-RNA). These findings were accompanied by reductions of SHIV-RNA and -DNA in lymphoid tissues as well as significantly improved survival in TIP-treated NHPs. Consistent with effective TIP replication and an R_0_ > 1, plasma TIP RNA was continuously detected in all treated animals over the observation period of 30 weeks.

This work provides a proof-of-concept for the use of defective viral genome-derived therapeutic interfering particles for the treatment of a chronic infection. If effective in inhibiting HIV-1 replication in humans, TIPs may reduce disease progression similar to how antiretroviral drugs do. Simulations based on the data observed in NHPs also indicated that TIP therapy may reduce the risk of HIV-1 transmission that is associated with the level of plasma viremia, although TIP effects on viral loads in bodily secretions remain to be determined (Fig. 1c). Continuous TIP detection in viremic animals and the capacity to establish reversible TIP latency suggest that HIV-targeting TIPs may provide persistent effects following a single administration. Although HIV-TIPs will not eliminate the viral reservoir (and neither do antiretroviral drugs), they provide an intriguing novel approach for controlling HIV-1 replication worth further investigation.

As the concept of TIPs for the treatment of HIV-1 infection advances from the bench to the bedside, critical considerations must be addressed (Fig. 1c). Continuous TIP replication and persistent integration into the human chromosome raise theoretical concerns about unwanted long-term effects, including potential insertional mutagenesis leading to lymphoid malignancies. Moreover, TIP/HIV-1 co-infection-mediated genetic recombination could lead to chimeric viral particle formation and TIP recombination with endogenous retroviral elements is a theoretical possibility, TIP replication might be associated with pathogenic immune activation or anti-TIP immunity, and HIV-1 evolution might result in escape from TIP-mediated interference. While no findings of concern appear to have been made over the 30-week observation period in TIP-treated NHPs, detailed safety analyses will be a particularly essential component for a first-in-human trial of HIV-TIPs. This includes the investigation of the risk of TIP transmission to other individuals. While this is a caveat urging ethical consideration, it has also been proposed as a way to mediate the spread of a therapeutic agent among people living with HIV (and is not entirely different from the potential spread of adjuvanted live vaccines).^4^ Finally, as the results of NHP experiments suggest, TIPs may not completely suppress HIV-1 plasma viremia. Residual HIV-1 replication may affect clinical and immunological outcomes and would be in contrast to highly effective antiretroviral therapy that (if available) commonly results in undetectable plasma HIV-1 RNA. Whether HIV-TIPs are effective in reducing viral rebound after interrupting antiretroviral therapy in vivo remains to be determined in preclinical and clinical studies.

Research into strategies to target HIV-1 has led to numerous remarkable scientific developments, including an antiretroviral drug that may provide near-universal protection when administered as infrequently as twice a year.^5^ While providing access to available effective antiretroviral drugs must be a cornerstone of global public health policy, novel approaches will likely be needed to curtail the HIV pandemic. Weinberger and colleagues have developed an innovative method aimed at reducing HIV-1 replication and transmission. Carefully conducted clinical trials will eventually be required to address safety considerations and investigate its efficacy and durability.

## References

[1] Pitchai, F. N. N. et al. Engineered deletions of HIV replicate conditionally to reduce disease in nonhuman primates. *Science* **385**, eadn5866 (2024). 39116226 10.1126/science.adn5866 PMC11545966

[2] Huang, A. S. & Baltimore, D. Defective viral particles and viral disease processes. *Nature* **226**, 325–327 (1970). 5439728 10.1038/226325a0

[3] Dimmock, N. J., Rainsford, E. W., Scott, P. D. & Marriott, A. C. Influenza virus protecting RNA: an effective prophylactic and therapeutic antiviral. *J. Virol.* **82**, 8570–8578, (2008). 18579602 10.1128/JVI.00743-08 PMC2519629

[4] Notton, T., Sardanyes, J., Weinberger, A. D. & Weinberger, L. S. The case for transmissible antivirals to control population-wide infectious disease. *Trends Biotechnol.* **32**, 400–405 (2014). 25017994 10.1016/j.tibtech.2014.06.006

[5] Bekker, L. G. et al. Twice-Yearly Lenacapavir or Daily F/TAF for HIV Prevention in Cisgender Women. *N. Engl. J. Med*. 10.1056/NEJMoa2407001 (2024). 10.1056/NEJMoa2407001 39046157

